# A 5G-powered robot-assisted teleultrasound diagnostic system in an intensive care unit

**DOI:** 10.1186/s13054-021-03563-z

**Published:** 2021-04-07

**Authors:** Shaobo Duan, Luwen Liu, Yongqing Chen, Long Yang, Ye Zhang, Shuaiyang Wang, Liuwei Hao, Lianzhong Zhang

**Affiliations:** 1 Henan Provincial People’s Hospital, Zhengzhou University People’s Hospital, No. 7, Weiwu Road, Jinshui District, Zhengzhou, 450003 Henan China; 2 Henan University People’s Hospital, Zhengzhou, 450003 Henan China

**Keywords:** Teleultrasound, Robot-assisted, Remote critical care medicine, Remote critical care ultrasound, 5G, Telemedical, Critical care medicine

## Abstract

**Background:**

Teleultrasound provides an effective solution to problems that arise from limited medical resources, a lack of local expertise, and scenarios where the risk of infection is high. This study aims to explore the feasibility of the application of a 5G-powered robot-assisted teleultrasound diagnostic system in an intensive care unit.

**Methods:**

In this study, the robot-assisted teleultrasound diagnostic system MGIUS-R3 was used. Using 5G network technology, the doctor manipulates the robotic arm to perform teleultrasound examination. The doctor can adjust parameters via the teleultrasound control panel, and real-time transmission of audio, video and ultrasound images can facilitate simultaneous communication between both parties. All patients underwent robot-assisted teleultrasound examination and bedside ultrasound examination of the liver, gallbladder, pancreas, spleen, kidney, as well as assessment for pleural effusion and abdominal effusion. We evaluated the feasibility of the application of the robot-assisted teleultrasound diagnosis system in the intensive care unit in terms of consultation duration, image quality, and safety. We also compared diagnostic consistency and differences.

**Results:**

Apart from one patient who was excluded due to severe intestinal gas interference and poor image quality, a total of 32 patients were included in this study. Every patient completed all relevant examinations. Among them, 20 patients were male; 12 were female. The average age of the patients was 61 ± 20 years. The average duration of teleultrasound diagnosis was 17 ± 7 min. Of the 32 patients, 26 had positive results, 6 had negative results, and 5 had inconsistent diagnoses. The overall diagnostic results were basically the same, and there were no differences in diagnostic levels between the two. The overall average image quality score was 4.73 points, which represented a high-quality image. After robot-assisted teleultrasound examination, no significant changes were observed in the vital signs of patients as compared to before examination, and no examination-related complications were found.

**Conclusion:**

The 5G-powered robot-assisted teleultrasound diagnostic system was associated with the benefits of clear images, simple operation, relatively high levels of consistency in terms of diagnostic results, higher levels of safety, and has considerable application value in the intensive care unit.

## Background

Telemedicine makes use of modern communication, electronic and computer technology to facilitate remote collection, storage, processing, transmission and querying of various medical information, in order to expand the scope of patient access to medical services by crossing geographical barriers, and is a discipline that provides clinical support for patients, thereby improving patient health [1]. Many studies have shown that remote consultations help to avoid the unnecessary long-distance transport of patients, which saves time and costs for the patient, and improves the quality of medical services [2–4]. The application of telemedicine in the intensive care unit can be traced back to the telemedicine consulting service implemented by Grundy et al. [5] in the 1980s. Studies have shown that when telemedicine technology is used in intensive care units, it can have a significant and positive impact on the prognosis of critically ill patients [6–8].

Ultrasound, an examination method that systematically shows the structure of organs, and which incorporates Doppler technology to show blood flow and measurement indicators, has become the core technology of critical care diagnosis and treatment and is widely used in intensive care wards [9, 10]. Point-of-care ultrasound (POCUS) is a non-invasive, non-toxic, and portable tool which has important clinical significance for evaluating critically ill patients, quickly identifying life-threatening diagnoses, and guiding invasive surgery in the intensive care unit [11]. However, traditional ultrasound requires the doctor/sonographer to carry equipment and wear isolation gowns when entering the intensive care and isolation ward for examination, which not only prolongs the consultation time, but also undoubtedly increases the potential risk of infection for medical staffs. The implementation of telemedicine provides a unique solution for critical care ultrasound examination.

In recent years, the development of computer networks, multimedia and communication technology has promoted the development of robot-assisted teleultrasound technology, and there has been an accumulation of abundant experience related to abdominal, heart, pelvic, obstetric, vascular and thyroid examinations [12]. The robot-assisted teleultrasound diagnostic system not only allows for dynamic observation with no radiation damage, but also reduces the risk of infection of medical staffs [13]. However, there are currently no feasibility studies on the application of a robot-assisted teleultrasound diagnostic system in intensive care units. This study explores the feasibility of implementing a 5G-powered robot-assisted teleultrasound diagnostic system in an intensive care unit.

## Methods

### Patients

All examinations were carried out with the informed consent of patients and their families, and were approved and supported by doctors and nurses. Inclusion criteria for patients: Patients diagnosed and treated in the intensive care unit of Henan Provincial People's Hospital from September to November 2020. Exclusion criteria for patients: (1) The patient’s condition is unstable, and doctors and nurses do not recommend the examination; (2) The patient’s abdomen is covered with accessory items and abdominal ultrasound cannot be performed.

### Instruments

The robot-assisted teleultrasound diagnostic system MGIUS-R3 was used in this study, equipped with ultrasonic probes: abdominal probe, model C5-1, frequency 2.5–5.0 MHz; shallow probe, model L15-4, frequency 5.0–12.0 MHz. The robot-assisted teleultrasound diagnostic system comprises a doctor's side and a patient's side. The components on the doctor's side include the main structure, operation control system, and audio and video system; the components on the patient's side are the main structure, motion execution system, ultrasound system, and audio and video system.

**Fig. 1:**
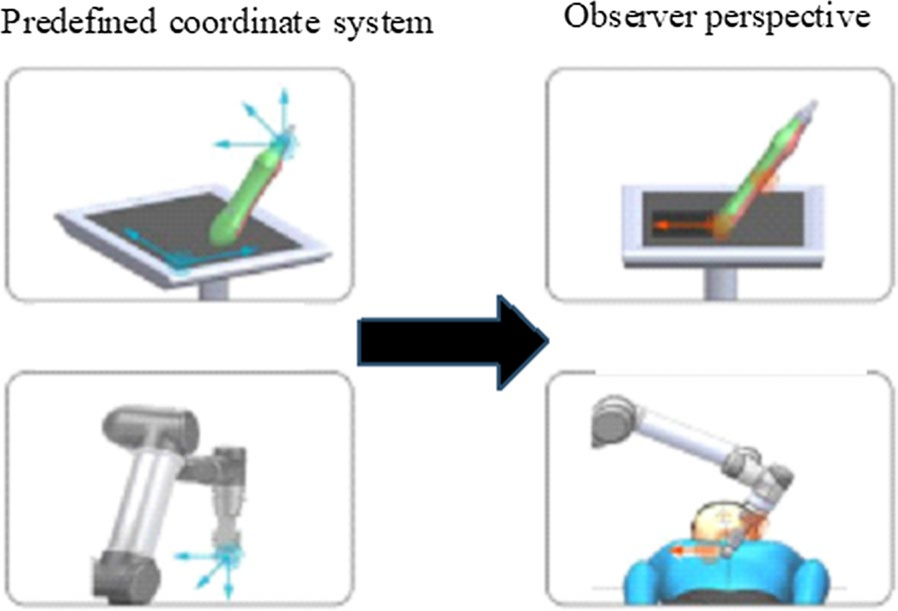
Schematic diagram of the remote control robotic arm. Through a series of coordinate system conversions and motion conversions, the motion of the operator is consistent with the motion of the robotic arm in the video display

**Fig. 2:**
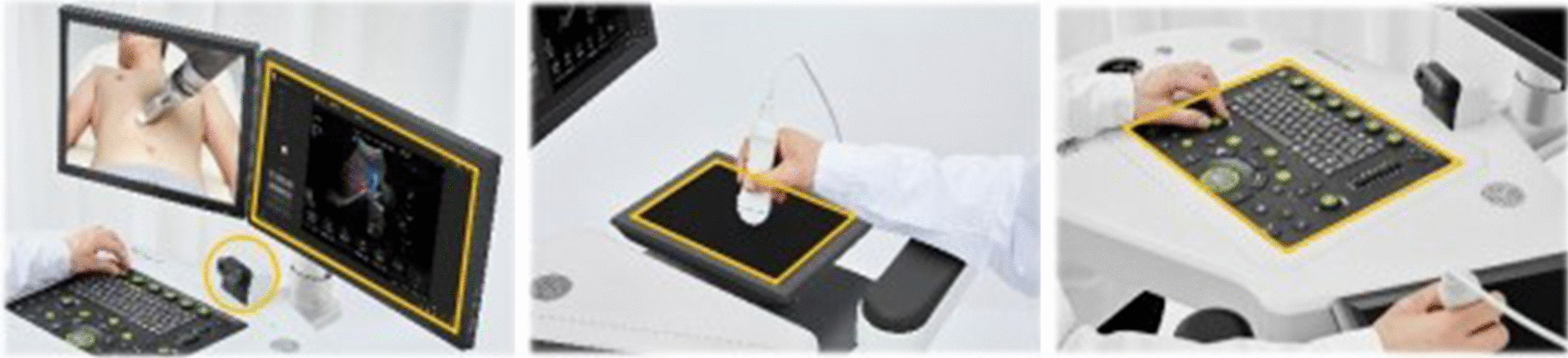
The figure shows the high-definition audio and video systems equipped on the doctor’s side (from left to right), which are used to transmit the doctor's voice and video; the simulated operator system is used to control the robotic arm; the teleultrasound control panel is used to adjust the parameters of the ultrasound machine

*Doctor's side* Through a predefined coordinate system, even if the operating system and the robotic arm are located in two different places, the robotic arm is controlled through a series of coordinate system conversions and motion conversions, so that the motion of the operator is consistent with the motion of the robotic arm in the video display from the observer's perspective (Fig. 1). The six-degrees-of-freedom simulated operating arm system with a force sensor could simultaneously control the posture and contact force of the robotic arm on the patient's side in real time, and the end-to-end delay was ≤ 200 ms. Via the teleultrasound control panel, the parameters of the ultrasound on the patient's side can be adjusted in real time, including frequency, gain, depth, parameter measurement, etc., and the end-to-end delay of remote parameter adjustment was less than 200 ms. Using a high-performance 1080P medical-grade display, ultrasound images could be read in real time, achieving high fidelity, low latency, and distortion-free subjectivity (Fig. 2). An ergonomic elbow rest is equipped next to the operation panel, and the doctor could place their elbow on the elbow rest during the ultrasound examination to relieve arm fatigue. The height adjustment was not less than 6 cm.

**Fig. 3:**
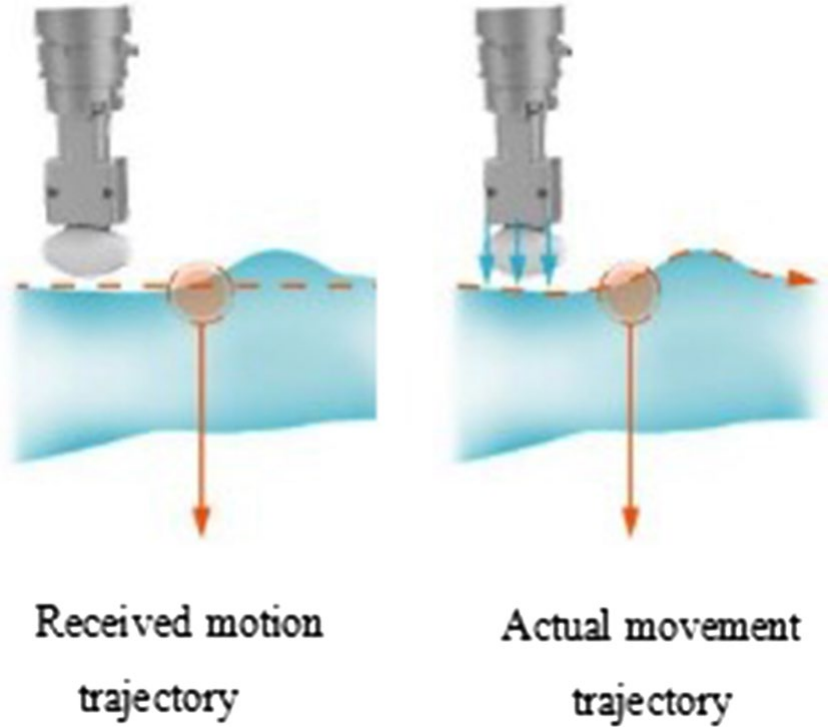
Schematic diagram of robotic arm movement. When the robotic arm is in contact with the human body, it moves smoothly along the contours of the skin

**Fig. 4:**
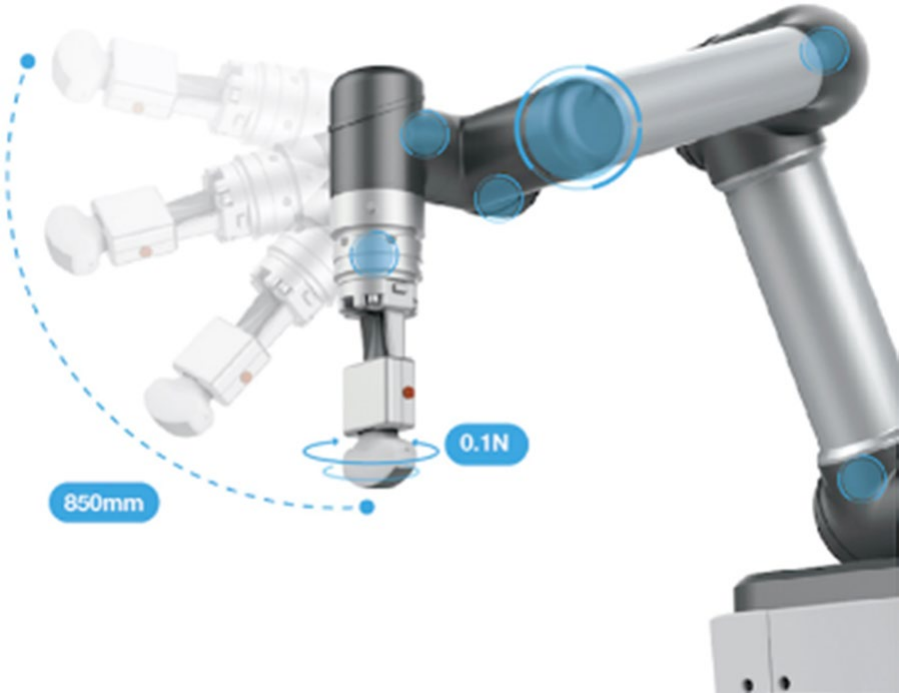
The robotic arm can perform six-dimensional motion in space, including three-dimensional rotation, two-dimensional planes, and one-dimensional force

*Patient end* The instrument is equipped with a flexible and controllable contact force control system and a six-axis collaborative robotic arm execution system. The robotic arm uses a flexible control algorithm to make the robotic arm move flexibly along the contours of the human body with an appropriate force during the contact between the robotic arm and the human skin surface (Fig. 3). The scanning coverage area of the robotic arm reaches 850 mm, the positioning accuracy of the system reaches ± 0.1 N, the force sensitivity is accurate to 0.1 N when stable, and the joint movement speed can reach 90°/s. The positioning sensor captures three-dimensional rotational movement, the position sensor captures two-dimensional plane movement, and the force sensor captures contact surface pressure. A total of six dimensions of data (three-dimensional rotation + two-dimensional plane + one-dimensional force) are thus collected, which can control the six-dimensional movement of the robotic arm in space (Fig. 4).

*Safety protection function* The mechanical arm has a maximum limit with regards to speed and pressure, and the force range of the mechanical arm (3–40 N) can be set by the doctor. The dual protection device uses a system design with an accurate force sensor and a reasonable force threshold, with the provision of "stop if excessive force + emergency stop button" to fully ensure patient safety.

Intelligent voice interaction: High-definition audio and video systems are equipped on both the doctor's side and patient's side, which can synchronize doctor-patient communication. The end-to-end delay of audio and video system interaction is less than 200 ms.

### Process establishment

**Fig. 5:**
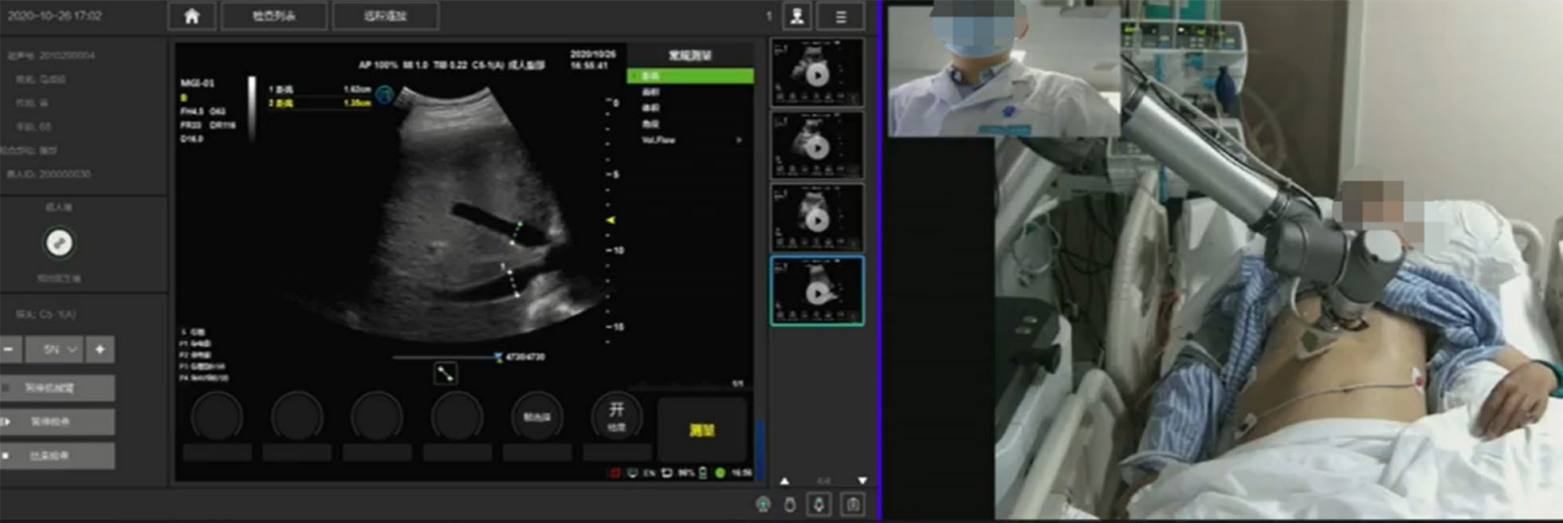
This is a real-time picture of robot-assisted teleultrasound diagnosis, which displays clearly the real-time transmitted ultrasonic images, the position of the probe on the patient and the patient's status

The robot-assisted teleultrasound diagnostic system is a real-time intelligent interactive system based on a 5G network (Fig. 5). The clinicians in the intensive care unit submit a consultation application in the teleultrasound diagnostic system based on an assessment of the patient's condition and needs. The application form includes basic information such as the patient's name, gender, age, and examination location. Once the request for consultation is received on the doctor's side, a remote connection can be initiated with the press of a key. The external environment of the patient and the ultrasound images collected by the robotic arm are transmitted to the doctor in real time through the 5G network for diagnosis. During the operation, the ultrasound doctor can complete the parameter adjustment of the ultrasound equipment on the patient side by controlling the ultrasound keyboard on the doctor side, and can also communicate with the patient side through audio and video systems of high quality. After the consultation, the ultrasound doctor promptly reports the examination results to the intensive care doctor.

### Information security

Databases and multiple encryption algorithms are used to protect user data privacy, in line with the EU GDPR Act and the requirements of various countries on data security.

### Internet

**Fig. 6:**
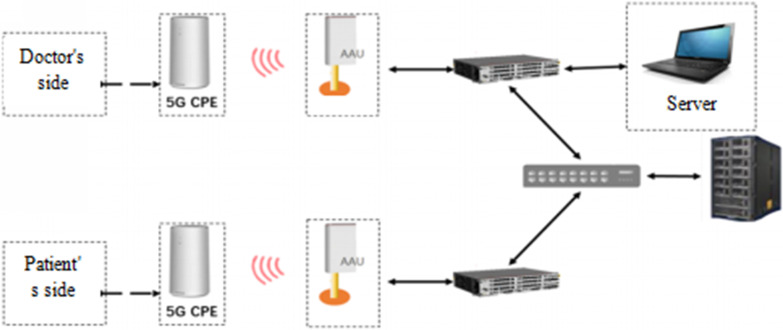
Relying on the advantages of 5G network's high bandwidth, wide connectivity, and low latency, robot-assisted teleultrasound diagnosis can be carried out smoothly without being restricted by the network

A 5G network was used for data transmission in this study. The connection method was as follows: doctor side-CPE-base station-server (operator dedicated line, fixed IP, open corresponding port)-base station-CPE-patient side (Fig. 6). The network download speed was 580 Mbps; the upload speed was 92 Mbps.

### Data collection

Because the general condition of patients in the intensive care unit was poor and they could not change body position as per the doctor's instructions, we performed partial examinations under limited conditions. Each patient received an examination of the liver, gallbladder, pancreas, spleen, and kidneys as well as a qualitative evaluation for pleural effusion and abdominal effusion. The examination results for pleural effusion and abdominal effusion were only recorded as "yes" or "no", and no positioning or quantitative analysis was performed. To investigate the accuracy of the diagnosis, we performed a second scan of the same site using bedside ultrasound. If there was an inconsistency in the results, a third ultrasound physician who was not present carried out an evaluation, and the result that was common to two parties was used. At the same time, after the robot-assisted teleultrasound examination was completed, the patient was evaluated to determine whether there were any obvious changes to vital signs and whether there were any complications related to the examination.

### Data analysis

The data were subjected to statistical analysis using Windows SPSS 18.0 K (SPSS Inc., Illinois, USA). The measurement data were expressed by $\overline{x}$ ± s. The paired sample t test and Kappa consistency test were used to evaluate the diagnosis results of remote ultrasound and bedside ultrasound. Kappa ≥ 0.75 indicated there was good consistency between the two; 0.75 > Kappa ≥ 0.4 indicated there was general consistency between the two; Kappa < 0.4 indicated poor consistency. A p value less than 0.05 was considered a statistically significant difference. In addition, we invited two off-site experts to use the subjective quality scoring method (MOS: Mean Opinion Score) to score the quality of the transmitted ultrasound images on the basis of an internationally prescribed 5-level absolute evaluation scale (5 points: No deterioration in the image quality is observed at all, very good; 4 points: a change in image quality can be seen but viewing is unhindered, good; 3 points: it can be clearly seen that the image quality has deteriorated, which hinders viewing slightly, fair; 2 points: viewing is hindered, poor; 1 point: viewing is severely hindered, very poor).

## Results

A total of 33 robot-assisted teleultrasound examinations were performed in this study. Among them, one patient was excluded due to severe intestinal gas interference and poor image quality. The remaining 32 patients underwent ultrasound examinations of the liver, gallbladder, pancreas, spleen, kidney as well as examinations for pleural effusion and abdominal effusion in accordance with the preliminary plan. Among them, 20 were male patients; 12 were female patients. The average age of the patients was 61 ± 20 years old, among which the oldest was 94 years old and the youngest was 13 years old. The longest time taken for remote ultrasound diagnosis was 37 min, the shortest was 9 min, and the average consultation time was 17 ± 7 min.

Of the 32 patients in this study, 26 had positive results and 6 had negative results. There were 5 cases with inconsistent diagnosis between the two parties (Table 1). Among them, there were 3 cases where there was a positive bedside ultrasound diagnosis but a missed diagnosis by robot-assisted teleultrasound, namely: 2 cases of liver cysts and 1 case of gallbladder polyp; and 2 cases with positive diagnosis via robot-assisted teleultrasound but missed diagnosis at the bedside, namely: 1 case of liver cyst, and 1 case of intrahepatic calcification. A total of 17 types of diseases were found in the positive results, of which the most frequently diagnosed were as follows: thick and rough gallbladder wall, 14 cases; gallbladder deposits, 12 cases; pleural effusion, 9 cases; abdominal effusion, 8 cases; liver cysts, 8 cases.

**Table 1:** Comparison of the results of robot-assisted teleultrasound diagnosis and bedside ultrasound diagnosis

	Bedside ultrasound		Total
	Negative	Positive	
Teleultrasound			
Negative	6	3	9
Positive	2	21	23
Total	8	24	32

The Chi-square test and the Kappa consistency test were used to perform statistical analysis on all data to evaluate the diagnostic differences and consistency between robot-assisted teleultrasound examination and bedside ultrasound examination (Table 2). In general, the McNemar value associated with the two inspection methods was close to 1, the Kappa value was 0.600, and the *p* value was < 0.001. The overall diagnosis results were basically the same, and there was no significant difference in the level of diagnosis. In addition, there was no significant difference in the diagnosis of 14 disease types using the two examination methods, and the level of consistency was high. The diagnosis of liver cysts was inconsistent in 3 cases. The McNemar value was close to 1, the Kappa value was 0.711, and the *p* value was less than 0.05, indicating that the diagnostic results of the two examination methods were essentially the same, and there was no difference in the level of diagnosis. Intrahepatic calcification and gallbladder polyps were diagnosed in 1 case with each examination, the McNemar value was close to 1, the Kappa value was 0.652, and the *p* value was less than 0.001, indicating that the diagnostic results associated with the two examination methods were basically the same, and there was no significant difference in the diagnosis level.

**Table 2:** Analysis of all positive diagnostic findings by different statistical methods

Positive diagnostic findings	Number of cases	Kappa value	*p* value
Gallbladder wall thickening	14	1.000	< 0.001
Gallbladder deposits	12	1.000	< 0.001
Pleural effusion	9	1.000	< 0.001
Abdominal effusion	8	1.000	< 0.001
Liver cyst	8	0.711	< 0.001
Fatty liver	5	1.000	< 0.001
Hyperechogenic liver	4	1.000	< 0.001
Enlarged gallbladder	4	1.000	< 0.001
Intrahepatic calcification	2	0.652	< 0.001
Gallbladder polyps	2	0.652	< 0.001
Cholecystitis	2	1.000	< 0.001
Right kidney cyst	2	1.000	< 0.001
Diffuse liver injury	1	1.000	< 0.001
Enlarged pancreas and uneven echogenicity	1	1.000	< 0.001
Pancreatic fat infiltration	1	1.000	< 0.001
Increased internal diameter of portal vein, inferior vena cava and hepatic vein	1	1.000	< 0.001
Bilateral hydronephrosis	1	1.000	< 0.001

On the basis of the internationally prescribed 5-level absolute evaluation scale, two transmission experts who were not present used a subjective quality scoring method to score ultrasound images that had been transmitted with the use of the 5G network. Among them, the number of cases with a score of 5 was greater than 70%. The average score of image quality provided by the two experts was greater than 4 points, and the total average score was 4.73 points, which represents a high-quality image (Table 3).

**Table 3:** Quality scoring of 5G-based teleultrasound diagnostic images

5G	Score					Total	Average score
	1 point	2 points	3 points	4 points	5 points		
Expert 1	0	0	1	6	25	32	4.75
Expert 2	0	0	0	9	23	32	4.71
Total	0	0	1	15	48	64	4.73

After the robot-assisted teleultrasound examination, the clinician in the intensive care unit evaluated the patient’s vital signs. All the vital signs of the patients showed no significant changes, and no complications related to the examination were found, which preliminarily demonstrates the safety of teleultrasound examination with the assistance of a robotic arm.

## Discussion

According to a report published by Subramanian et al. [14] in <Critical Care Medicine> in 2020, two-thirds of remote intensive care centers use remote radiology to assist in the management of patients; however, they did not specifically mention the role of teleultrasound in the management of critically ill patients [15]. In fact, from a practical perspective, for critically ill patients on an isolation ward, CT examination is not only inconvenient because the patient has to be transported to the imaging area, but it also increases patient exposure to radiation, which is not conducive to repeated dynamic observations; furthermore, the difficulty associated with CT scanner disinfection increases the possibility of cross infection [13]. Teleultrasound is a kind of telemedicine science that transmits ultrasound images of difficult cases in remote areas to higher-level hospitals through the network, whereby consultation experts can provide a diagnosis as well as analysis for decision-making. It provides effective solutions to the problems arising from limited medical resources, a lack of local expertise, and situations involving a high risk of infection [16]. In this study, through the application of 5G network technology, 32 ultrasound examinations were completed with the use of a robot-assisted teleultrasound diagnostic system. Whether considered from the perspective of network transmission speed, image quality, or consistency with bedside ultrasound results, the findings in this study demonstrated that robot-assisted teleultrasound diagnosis has a relatively high level of feasibility in the intensive care unit and provides a novel solution for the application of ultrasound in the intensive care unit.

It has been shown that the sensitivity, specificity, and accuracy of ultrasound in the diagnosis of pleural effusion are about 84%, 100%, and 94%, respectively, and the results are equivalent or superior to routine chest X-ray examinations in a series of surgical ICU patients [17]. Ultrasound is similarly applicable to the diagnosis of abdominal effusion. We used robot-assisted teleultrasound to diagnose pleural effusion and abdominal effusion, and 9 cases with pleural effusion and 8 cases with abdominal effusion were identified. The results of teleultrasound diagnosis were 100% consistent with the results of bedside ultrasound examination results, with no differences observed. However, since most patients in the intensive care unit could only remain in the supine position and could not stand, lie on their side or get up to facilitate the localization of pleural effusion during examinations, we only performed a qualitative diagnosis in this study, and did not perform localized diagnosis. This can be a direction for future development with regards to the application of robot-assisted teleultrasound in intensive care units.

For critically ill patients in the intensive care unit, acalculous cholecystitis is a very common condition in critical care medicine. The atypicality of clinical symptoms increases the difficulty of assessment, but ultrasound can provide additional information to enable a diagnosis to be made [18]. Ultrasound manifestations of acalculous cholecystitis are as follows: gallbladder volume enlargement (short axis diameter > 40 mm), wall thickness (> 3 mm), or "bilateral signs", with flocculent deposits in the gallbladder. Combined with clinical laboratory indicators, the diagnosis can be made. In this study, 2 patients had the above-mentioned ultrasound findings, and there were no ultrasound signs of stones or polyps. It is therefore recommended that intensive care physicians combine clinical indicators for further diagnosis. In these two patients, the results of teleultrasound diagnosis were consistent with those of bedside ultrasound. The diagnostic results were consistent, and there was no significant difference in the level of diagnosis. In the remaining examinations, two cases of hepatic cysts and one case of gallbladder polyp were missed in teleultrasound diagnosis, while one case of liver cyst and one case of intrahepatic calcification were missed by bedside ultrasound. In general, the Kappa value of the two examination methods was 0.600, and the *p* value was < 0.001, indicating that the overall diagnostic results associated with the two methods were basically the same, and there was no significant difference in the diagnosis level.

In the early development of robot-assisted teleultrasound, the quality of ultrasound images was limited by network bandwidth, and the image display was not very clear [19, 20]. With advancements in network technology, however, the image quality transmitted by the robot-assisted teleultrasound diagnostic system has improved. It has been shown that the image quality associated with this method is comparable to that of traditional ultrasound [21, 22]. Compared with 4G network technology, the transmission rate of the latest 5G network communication technology is 20 times better, the mobile speed is 1.5 times better, and the delay is reduced by a factor of 10. It has high bandwidth, low delay, and wide connectivity, and can fully support the transmission of multi-channel high-definition video, accurate real-time transmission of ultrasound images, and at the same time enable the networking of a large amount of medical equipment inside and outside the hospital. The ultrasound image obtained by the 5G-powered robot-assisted teleultrasound diagnostic system was evaluated using an internationally standardized subjective quality scoring method. The average score with regards to image quality was 4.73 points, which represents a high-quality image, surpassing the limitations of previous networks regarding ultrasound image transmission, thereby meeting the requirements of teleultrasound diagnosis.

A 2017 study by Scott J. Adams et al. demonstrated that it is feasible to use a robot-assisted teleultrasound system for adult abdominal examinations. However, their system had technical limitations. The sonographer could not control the pressure and movement of the probe, resulting in the need for an on-site assistant on the patient side to position the robotic arm and apply pressure on the patient's abdomen [23]. Our robot-assisted teleultrasound diagnostic system could be started by remote connection at the press of a button, thus directly initiating the mechanical arm, which moves flexibly along the contours of the human body with appropriate pressure. The mechanical arm had a maximum speed and pressure limit, and the force of the mechanical arm could be set by the doctor in the range of 3–40 N. In our study, the teleultrasound control panel could be used to achieve remote adjustment of the parameters of the patient's ultrasound, including frequency, gain, depth, parameter measurement, etc. In addition, this system had dual protection devices and a function called "stop if excessive force + emergency stop button", which helped to fully ensure patient safety. In the 2019 novel coronavirus disease (COVID-19) crisis, high-resolution CT (HRCT) imaging was widely used in the diagnosis of COVID-19 due to its high spatial resolution. However, potential hazards still remain with regards to this procedure [12, 13]. At present, increasing evidence shows that ultrasound plays an important role in the diagnosis and treatment of COVID-19 patients, and provides an important basis for formulating treatment strategies [24, 25]. A number of studies have used 5G-powered robot-assisted teleultrasound diagnostic systems to achieve effective ultrasound assessment for evaluating the cardiopulmonary function of COVID-19 patients. Preliminary results show that the protocol is feasible [12, 13, 26, 27]. But the sample sizes in these studies were small and the results lacked statistical significance. This study was based on a 5G-powered robot-assisted teleultrasound diagnostic system, and its preliminary application in the intensive care unit was investigated, which demonstrated its feasibility, providing a better foundation for the medical management of patients in intensive care units during a possible future epidemic.

This research preliminarily demonstrates the feasibility of applying a 5G-powered robot-assisted teleultrasound diagnostic system in an intensive care unit. But there are limitations. First of all, although the mechanical arm can achieve movement with six degrees of freedom, it still has restrictions on the side. It is necessary to manually move the position of the machine away from the bed to achieve the desired direction. Secondly, the system currently lacks a cardiac probe and cannot perform scans of the heart. In addition, the operating doctors in this study were all senior ultrasound experts, and we did not investigate whether less experienced ultrasound doctors could also carry out the examination easily. Finally, robotic tele-operation is indeed a certain challenge for remote experts in terms of 3D space perception. Doctors need to repeatedly practice and familiarize themselves with the operation process, so as to realize the "human–machine integration" between the operator and the free arm.

## Conclusion

The application of a 5G-powered robot-assisted teleultrasound diagnostic system in the intensive care unit was associated with the benefits of ease of operation, a simple process, clear images, essentially comparable diagnostic results to bedside examination, no significant difference in diagnostic level, high levels of safety, a reduction in the risk of infection for both the patient and the ultrasound physician, and good feasibility, which deserves to be used widely in clinical practice.

## Data Availability

The datasets used and/or analyzed during the current study are available from the corresponding author on reasonable request.
